# A multipredictor model to predict the conversion of mild cognitive impairment to Alzheimer’s disease by using a predictive nomogram

**DOI:** 10.1038/s41386-019-0551-0

**Published:** 2019-10-21

**Authors:** Kexin Huang, Yanyan Lin, Lifeng Yang, Yubo Wang, Suping Cai, Liaojun Pang, Xiaoming Wu, Liyu Huang, for the Alzheimer’s Disease Neuroimaging Initiative

**Affiliations:** 1 School of Life Science and Technology, Xidian University, Xi’an, Shaanxi 710071 P. R. China; 2 The Key Laboratory of Biomedical Information Engineering of Ministry of Education, School of Life Sciences and Technology, Xi’an Jiaotong University, Xi’an, 710049 P. R. China

**Keywords:** Cognitive ageing, Predictive markers, Cognitive ageing, Predictive markers

## Abstract

Predicting the probability of converting from mild cognitive impairment (MCI) to Alzheimer’s disease (AD) is still a challenging task. This study aims at providing a personalized MCI-to-AD conversion estimation by using a multipredictor nomogram that integrates neuroimaging features, cerebrospinal fluid (CSF) biomarker, and clinical assessments. To do so, 290 MCI patients were collected from the Alzheimer’s Disease Neuroimaging Initiative (ADNI), of whom 76 has converted to AD and 214 remained with MCI. All subjects were randomly divided into a primary and validation cohort. Radiomics signature (Rad-sig) was obtained based on 17 cerebral cortex features selected by using Least Absolute Shrinkage and Selection Operator (LASSO) algorithm. Clinical factors and amyloid-beta peptide (Aβ) concentration were selected by using Spearman correlation between the converted and not-converted patients. Then, a nomogram that combines image features, clinical factor, and Aβ concentration was constructed and validated. Furthermore, we explored the associations between various predictors from the macro- to the microperspective by assessing gene expression patterns. Our results showed that the multipredictor nomogram (C-index 0.978 and 0.956 in both cohorts, respectively) outperformed the nomogram using either Rad-sig or Aβ concentration as individual predictors. Significant associations were found between neuropsychological scores, cerebral cortex features, Aβ levels, and underlying gene pathways. Our study may have a clinical impact as a powerful predictive tool for predicting the conversion probability of MCI and providing associations between cognitive impairment, structural changes, Aβ levels, and underlying biological patterns from the macro- to the microperspective.

## Introduction

Alzheimer’s disease (AD) remains an irreversible neurodegenerative condition characterized by progressive cognitive and memory impairments, which have a vicious influence on an individual’s daily life and the social healthcare system [1]. Since current drug therapies cannot directly prevent the progression of AD, more hope has been placed on early prediction of AD [2]. Related to this, mild cognitive impairment (MCI), which is usually regarded as an intermediate stage between normal aging and AD, is a potential target for predicting individuals at risk of developing AD [3]. Moreover, studies have shown that treatment decisions would greatly benefit from early diagnosis, which may delay the progression of AD [4–7]. Therefore, accurately predicting and identifying the probability of deterioration in MCI patients is a pressing need.

Several potential biomarkers have been identified as useful predictors for early AD prediction, such as structural brain changes, disrupted functional connectivity, and tau protein and amyloid-beta plaque accumulation [8–11]. However, these biomarkers often focus on one or several different aspects of AD progression, and few studies have attempted to explore the power of combining predictors from different aspects, which may provide more precise information about risk assessment.

Hence, a simple, accurate, and reliable method to assist in clinical prediction that considers several risk factors is needed to refine the prognosis of patients with AD progression. Nomograms, an emerging method for the support of precise clinical decisions, combines several indicators rather than an analysis of individual factors based on multivariable logistic analysis [12–14]. Moreover, nomograms can predict individualized specific risk for each patient [15]. In this study, we aimed to (1) select significant predictors from structure imaging findings, cerebrospinal fluid (CSF) markers, and clinical pathology using different strategies; (2) build a multipredictor nomogram in a primary cohort; and (3) validate the predictive power of the nomogram in an independent cohort.

In addition, we noted that there may be associations that link these predictors from the macro- to the microperspective, and underlying biological patterns such as gene pathways might reflect potential pathological information at the microlevel in AD progression. We explored the associations between these predictors by adding gene expression patterns. We expect this research to provide a powerful predictive tool for predicting the conversion probability of MCI to AD and to provide new information on manifestation-to-molecular associations in neurodegeneration.

## Materials and methods

### Participants

All data were obtained from the Alzheimer’s Disease Neuroimaging Initiative (ADNI) database, including ADNI-1, ADNI-GO, and ADNI-2 studies (http://adni.loni.usc.edu/). MCI patients with a follow-up period of at least 6 months were considered eligible for this study. Furthermore, we restrict our selection to the MCI patients with a complete 3T magnetic resonance image (MRI) scan and related neuropsychological assessments. The selected patients were divided into two groups based on their Clinical Dementia Rating (CDR) scores: converters (MCI_C), whose first diagnosis of MCI (baseline CDR = 0.5) changed to AD (final CDR = 1) at the latest diagnosis, and nonconverters (MCI_NC), whose diagnoses did not change and the CDR scores remained as 0.5 at the latest diagnosis. Totally, we have selected 290 MCI patients. Among them, 76 patients had converted to AD, and other 214 patients remained at MCI at their last entry. Two-thirds of all patients (50 MCI_C patients and 141 MCI_NC patients in the primary cohort) were used for feature selection and nomogram training. The remaining one-third of the patients, including 26 MCI_C patients and 73 MCI_NC patients were used for validating the selected features and nomogram as validation cohort.

### Image acquisition

In this study, standard T1-weighted anatomical imaging was obtained by volumetric three-dimensional magnetization-prepared rapid gradient-echo (3D-MPRAGE) or equivalent protocols with slightly different resolutions across patients. Only 3 T MRI images were utilized in the validation cohort to remain consistent with the primary cohort. The detailed imaging protocols are provided at ADNI website (http://adni.loni.usc.edu/methods/documents/).

### Data preprocessing and image feature extraction

The preprocessing and feature extraction processes were conducted by Freesurfer (http://surfer.nmr.mgh.harvard.edu/). Freesurfer is a software package for the analysis and visualization of structural MRI images that we used to extract cortical features in this study. Preprocessing included the following: motion correction, skull stripping, coordinate transformation, gray–white matter segmentation, reconstruction of cortical surface models, region labeling, registration, and statistical analysis [16–19]. This process was conducted with the “recon-all” script, and all settings were held at the default values.

### Image feature selection and radiomics signature construction

The image features were selected using a radiomics strategy. The term radiomics has recently attracted increased discussion in medical imaging research and refers to transforming medical images into high-dimensional data and extracting significant features by data-characterization algorithms. Advances in these machine-learning methods have facilitated the development of medical data mining, enabling personalized predictions, and improving predictive accuracy. In our study, the Least Absolute Shrinkage and Selection Operator (LASSO) method was conducted to select significant features from Freesurfer between MCI_C and MCI_NC patients in the primary cohort. LASSO is a robust method that is especially suitable for the regression of high-dimensional features in a radiomics strategy. The radiomics signature (Rad-sig), which was defined as a linear combination of the selected significant features with their weighted coefficients provided by LASSO, was regarded as a predictor of structural brain changes.

### Assessment and validation of the radiomics signature

The support vector machine (SVM) was used prior to nomogram construction to validate the effectiveness of selected image features based on Rad-sig. In particular, tenfold cross-validation, which was applied on the primary cohort, can provide a reliable estimation of the usefulness of the Rad-sig feature. Accuracy and the receiver-operating characteristic (ROC) curve were used to represent the performance of the selected features. We calculated the average accuracy and ROC after tenfold cross-validation as the measure of performance in the primary cohort. Then, we validated the feature performance in the validation cohort.

### Collection and selection of clinical and CSF indicators

Clinical indicators, including demographic information and neuropsychological scale scores, were obtained from the ADNI assessment files (http://ida.loni.usc.edu/pages/access/studyData.jsp?categoryId=12). The demographic information included age, sex, and education level for all patients. Furthermore, we collected the scores from six neuropsychological scales, including the Functional Activities Questionnaire (FAQ), the Alzheimer’s Disease Assessment Scale (ADAS, both 13 and 11 questionnaires), the Mini-Mental State Examination (MMSE), the Neuropsychiatric Inventory Questionnaire (NPI-Q), and the Geriatric Depression Scale (GDS), as candidate clinical predictors [20–24].

The CSF indicators collected from the ADNI database were the concentrations of the amyloid-beta peptides (Aβ) in CSF aliquot samples, which were analyzed by 2D-UPLC-tandem mass spectrometry in the UPenn ADNI Biomarker Core laboratory (https://ida.loni.usc.edu/pages/access/studyData.jsp?categoryId=11&subCategoryId=33). Aβ, which has been recently considered as one of the core neurobiological factors in AD progression [25]. 2D-UPLC-tandem mass spectrometry is a reliable method that can provide accurate results for peptide levels [26]. Each value for a patient was the average of analyses of duplicate 0.1 -mL aliquots from each CSF sample.

Statistical analyses were conducted for both the clinical and CSF indicators. In this study, we calculated Spearman correlation of the scores or concentrations between the patient status (converted = 1, stable = 0) to select the predictors that were most relevant to MCI conversion.

### Development and assessment of a multipredictor nomogram

A nomogram is a graphical calculating device, a two-dimensional diagram designed to allow the approximate graphical computation of a mathematical function. A multipredictor nomogram could visualize the results of logistic regression or cox regression with several predictors. For each patient, it calculates a total score by summing up all scores of predictors, and obtain the probability of occurrence of each patient event by a conversion function between the score and the probability. In this study, the calculated Rad-sig, selected clinical measures, and CSF biomarkers were separately entered into the nomogram as individual predictor. Then, we combined those predictors to built a multipredictor nomogram.

We used the primary cohort to construct the nomogram and used the validation cohort to assess the performance of the constructed predictive nomogram. To obtain a more reliable model, bootstrapping validation with 1000 resampling was conducted to overcome the overfitting problem. The predictive power was measured by the concordance index (C-index), which ranges from 0.5 to 1. A higher value represents a higher predictive accuracy [27]. The calibration curve provided a comparison between the expected and observed conversion probabilities. The whole process was performed in R 3.3.2 (http://www.r-project.org/).

### Association analysis of predictors from macro- to microperspective

**Fig. 1:**
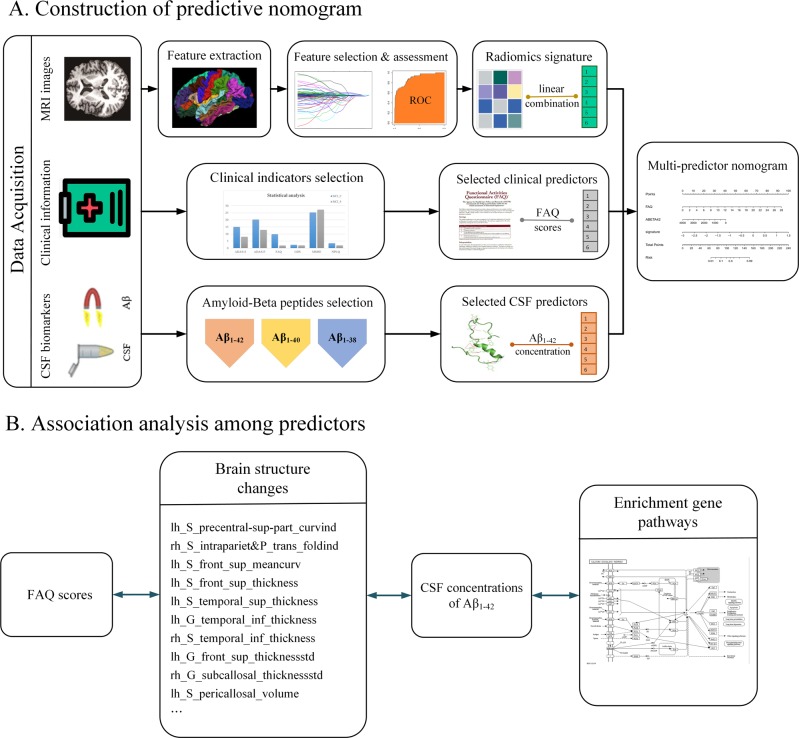
The flow diagram for the whole study process. **a** The construction of nomogram. **b** Association analysis of predictors by adding gene expression patterns

To explore the causal synergy in the pathophysiology, we calculated Spearman correlations with SPSS 23.0. The FAQ score, which usually reflects disease degree and clinical manifestations of the patients, may be associated with changes in the cerebral cortex. The abnormal Aβ concentration may be one of the factors causing structural changes. To further analyze the underlying genetic mechanisms that may be associated with the concentration of Aβ, we added RNA gene expression data (http://ida.loni.usc.edu/pages/access/geneticData.jsp) into the chain of association. The gene expression profile for 49,395 gene transcripts was analyzed on Affymetrix chips and obtained from the ADNI database. Differential expression analysis and enrichment analysis between MCI_C and MCI_NC patients were conducted by the Database for Annotation, Visualization and Integrated Discovery (DAVID) v6.8 (https://david.ncifcrf.gov/) to enrich the significant biological pathways in the Kyoto Encyclopedia of Genes and Genomes (KEGG) pathway database. In this study, we summed the expression values of the differentially expressed genes in a significant pathway and defined this value as the pathway expression indicator. Then, we identified associations between underlying gene pathways and Aβ concentrations. Figure 1a shows the prediction process of this study, while Fig. 1b shows the process of the association analysis.

## Results

### Demographic and clinical characteristics

As shown in Table 1, data from a total of 290 MCI patients were collected from the ADNI database, including 76 MCI converters (MCI_C) and 214 MCI nonconverters (MCI_ NC). We randomly divided them into the primary cohort (*n* = 191) and validation cohort (*n* = 99). The average age of the patients was 72.53 years in the primary cohort, and 71.73 years in the validation cohort. The overall percentage of men was 55.5% (161 of 290). The average education level of all patients was 16.11 years. There were no significant differences between the MCI_C and MCI_NC in age and education level in either the primary or validation cohorts.

**Table 1:** Characteristics of MCI patients in the primary cohort and validation cohort

	Primary cohort (*n* = 191)		*P*-value	Validation cohort (*n* = 99)		*P*-value
Characteristics	MCI_C (*n* = 50)	MCI_NC (*n* = 141)		MCI_C (*n* = 26)	MCI_NC (*n* = 73)	
*Demographic information, mean (SD), years*						
Age	73.22 (7.29)	72.29 (7.374)	0.444	74.85 (6.488)	70.62 (7.72)	0.293
Sex (M/F)	26/24	80/61	–	13/13	42/31	–
Education level	15.85 (2.84)	16.28 (2.69)	0.671	15.85 (2.84)	16.04 (2.65)	0.753
*Amyloid-Beta peptides in CSF aliquot samples, mean (SD), pg/mL*						
Aβ_1–42_	829.12 (301.55)	1239.38 (587.0)	0.000	691.69 (217.76)	1312.6 (644.15)	0.000
Aβ_1–40_	7878.66 (1983.14)	8525.8 (2482.07)	0.098	8368.23 (2370.74)	8256.6 (2587.40)	0.340
Aβ_1–38_	1835.54 (487.50)	1949.06 (574.48)	0.214	1948.96 (622.44)	1884.01 (596.35)	0.639
*Neuropsychological scales, mean (SD)*						
ADAS11 score	17.5 (5.89)	7.89 (3.26)	0.000	19.10 (8.85)	7.53 (3.18)	0.000
ADAS13 score	27 (7.39)	12.66 (5.25)	0.000	28.53 (10.85)	12.37 (5.75)	0.001
CDR score (baseline)	0.5	0.5	–	0.5	0.5	–
CDR score (latest)	1	0.5	–	1	0.5	–
FAQ score	14.16 (6.31)	2.1 (3.37)	0.000	11.12 (5.95)	1.73 (2.94)	0.000
GDS score	2.38 (2.56)	1.74 (1.39)	0.029	2.92 (2.29)	1.83 (1.64)	0.162
MMSE score	24.68 (5.91)	28.40 (1.53)	0.000	23.38 (3.38)	28.38 (1.48)	0.000
NPI-Q score	4.72 (4.07)	2.09 (3.02)	0.004	4.00 (4.71)	2.31 (2.86)	0.014

*MCI_C* the converter group, *MCI_NC* the stable group, *CSF* cerebrospinal fluid, *Aβ*_*1–42*_ amyloid-beta 1–42, *Aβ*_*1–40*_ amyloid-beta 1–40, *Aβ*_*1–38*_ amyloid-beta 1–38, *SD* standard deviation, *ADAS* Alzheimer’s Disease Assessment Scale (with 11 and 13 questionnaires, respectively), *CDR* clinical dementia rating, *FAQ* functional activities questionnaire, *GDS* geriatric depression scale, *MMSE* mini-mental state examination, *NPI-Q* neuropsychiatric inventory questionnaire

The concentrations of Aβ peptides in CSF and neuropsychological scale scores are also shown in Table 1. Levels of three Aβ peptides, including Aβ_1–42_, Aβ_1–40_, and Aβ_1–38_, which have been indicated to be related to AD, were provided by the UPenn laboratory. However, only Aβ_1–42_ showed a significant difference between the MCI_C and MCI_NC groups according to Student’s *t* test. All neuropsychological scale scores showed significant differences between the two groups (*P* < 0.05).

### Extraction of image feature and construction of the radiomics signature

**Fig. 2:**
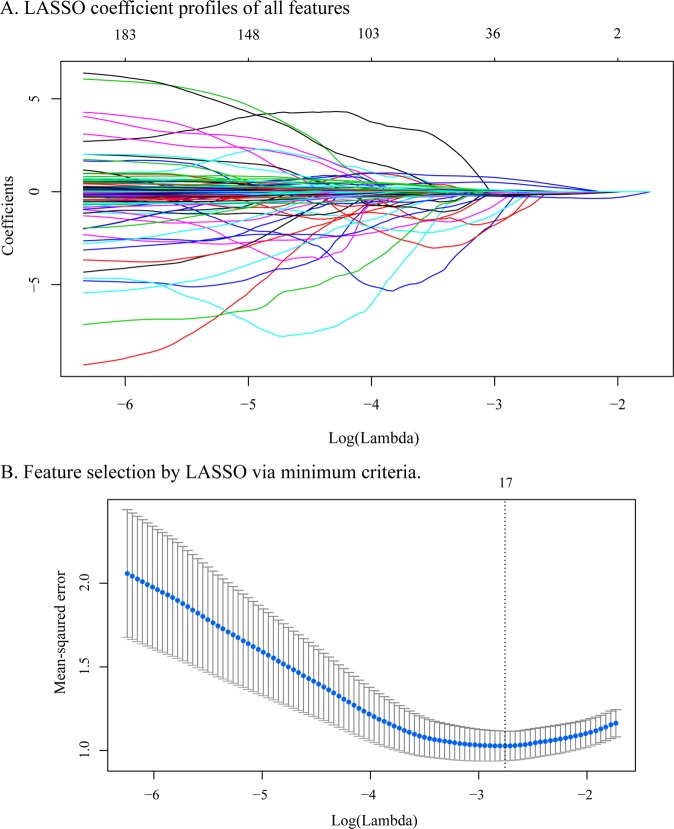
Feature selection using the LASSO binary logistic regression model. **a** LASSO coefficient of the total 1036 features. A coefficient profile plot was provided against the log (Lambda) sequence. **b** Feature selection in the LASSO model used tenfold cross-validation via minimum criteria. Blue-dotted vertical lines were drawn at the optimal values by using the minimum criteria (minimize the mean-squared error), the value 17 represents that 1036 features were reduced to 17 nonzero features by LASSO

Image features were extracted by Freesurfer, which is a tool that can calculate cortical indicators, including average thickness, standard deviation of thickness, integrated rectified Gaussian curvature, integrated rectified mean curvature, intrinsic curvature index, folding index, and gray matter volume. In this study, to obtain more detailed features to enhance the prediction accuracy, we used the Destrieux atlas, which divided the whole cortex into 148 regions. Then, a feature set with a total of 1036 features (148 × 7 = 1036) was used. Feature selection was conducted by LASSO regression in the primary cohort. Each feature has a coefficient as its weight provided by LASSO, as shown in Fig. 2a. When the binomial deviance was minimized, 17 significant features, i.e., image predictors, were selected from among all the features. Figure 2b shows the feature selection process by LASSO. The Rad-sig was a linear combination of the selected features and their coefficients. The full name and abbreviations of the selected features, as well as the Rad-sig calculation formula, are shown in Supplementary Material 1.

### Assessment and validation of the radiomics signature

The SVM classifier based on the radial basis function kernel estimated the performance of the Rad-sig. The accuracy of the SVM classifier in the primary cohort was 86.4%, and the mean area under the curve (AUC) after tenfold cross-validation was 89.6%. To further verify the classification effect of the Rad-sig, we also used the SVM classifier to classify the data in the validation set. The accuracy was 80.0%, and the AUC was 84.6%. Figure which provided in the Supplementary Material 3 shows the ROC curve in the primary and validation cohorts in black and red, respectively.

### Selection of the clinical and CSF predictors

We calculated Spearman correlations between the indicators and patients’ status. As shown in Supplementary Material 2, the FAQ score was the most significant indicator with the largest correlation coefficients among all clinical indicators, and Aβ_1–42_ was the only indicator with a significant correlation with patient status. As shown in Table 1, according to *t* tests, there were also significant differences in the FAQ scores and Aβ_1–42_ concentrations between the MCI_C and MCI_NC groups. Hence, in this study, we chose the FAQ scores and Aβ_1–42_ concentrations as predictors in the nomogram.

### Estimation of the performance of the individual predictors

We estimated the performance of the Rad-sig, FAQ scores and Aβ_1–42_ CSF concentrations as individual predictors, and the results are shown in Supplementary Material 3. The performance of all three predictors was good in predicting MCI conversion. The predictive accuracy of the FAQ scores was the highest among all predictors, with a C-index of 0.921 in the primary cohort and 0.910 in the validation cohort. The Rad-sig had a C-index of 0.90 in the primary cohort, and 0.869 in the validation cohort. The C-index of the Aβ_1–42_ concentrations was 0.769 and 0.831 in the primary and validation cohorts, respectively.

### Construction and validation of the multipredictor nomogram

**Fig. 3:**
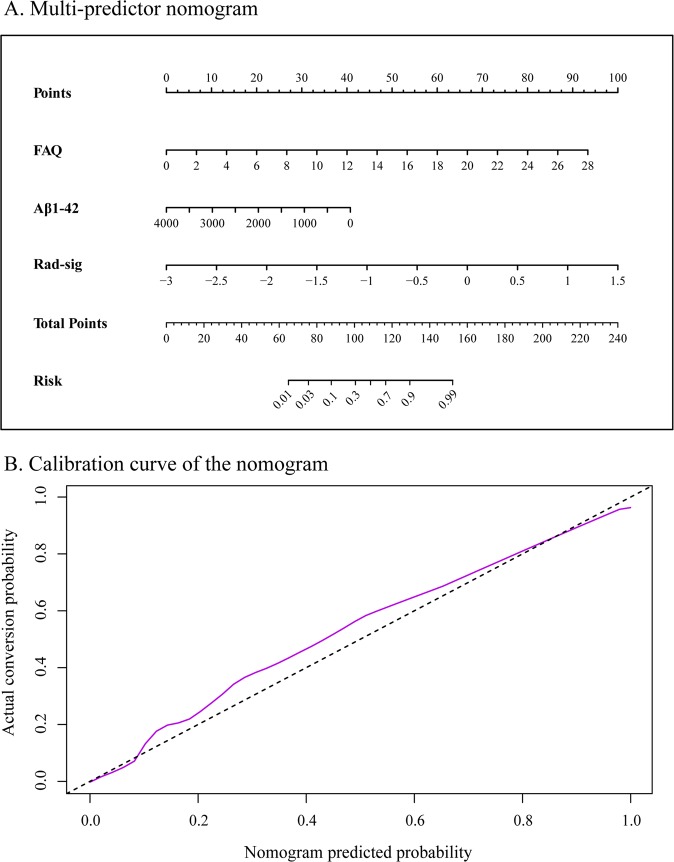
**a** Predictive nomogram integrates the functional activities questionnaire (FAQ), concentration of the amyloid-beta peptides (Aβ) in CSF aliquot samples and the radiomics signature based on selected features. **b** Calibration curve of the nomogram. Calibration curve represents the calibration of the nomogram, which shows the consistency between the predicted probability of conversion and actual conversion probability of MCI patients. The *x*-axis is the predicted probability by nomogram and the *y*-axis is the actual conversion rate of MCI patients. The black-dotted line represents a perfect prediction by an ideal model, and the purple solid line shows the performance of the nomogram, of which a closer fit to the dotted line means a better prediction

The nomogram that combined the three significant predictors was constructed. Figure 3a shows the predictive nomogram developed in the primary cohort, which obtained a C-index of 0.978 (95% CI, 0.960–0.995). Figure 3b shows the calibration curve of the predictive nomogram. The closer the calibration curve is to the diagonal, the better the predictive power of the nomogram. Then, we validated the nomogram in the validation cohort, and the C-index was 0.956 (95% CI, 0.919–0.992). The performance of the nomogram which combined multiple factors was significantly increased as compared to the model used only individual predictor (*P* < 0.05 in both primary and validation cohorts).

### Association analysis

**Fig. 4:**
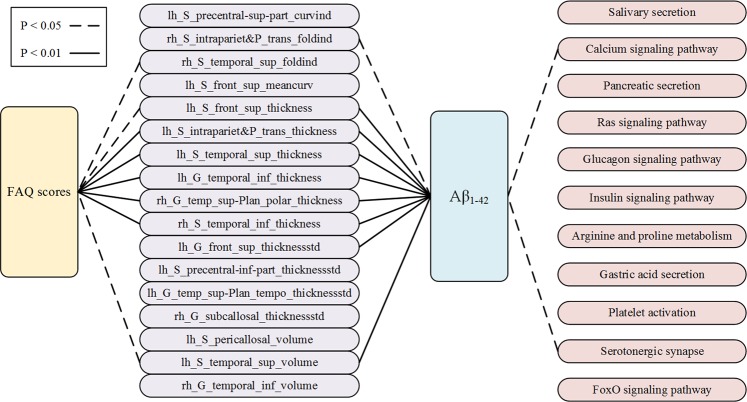
Association analysis between the FAQ scores, 17 image features, concentration of Aβ_1–42_, and 11 enriched gene pathways in the validation cohort. The solid lines represent strong significant association between factors (*P* < 0.01) and the dotted lines represent significant association (*P* < 0.05)

Furthermore, an association study was conducted to delineate the relationship between the features employed in nomogram construction and gene expression pattern. Our results showed that there exists a significant association between the FAQ scores and the cortical anatomic changes. As shown in Fig. 4, there were significant associations between the FAQ scores and the thickness of the superior frontal sulcus, the intraparietal and transverse parietal sulci, the superior temporal sulcus and the inferior temporal sulcus in the left hemisphere, as well as the thickness of the planum polare of the superior temporal gyrus and the inferior temporal sulcus in the right hemisphere. The folding index of the right superior temporal sulcus and the gray matter volume of the left superior temporal sulcus also showed correlations to the FAQ scores. Especially, we found the cortical thickness and gray matter volume which correlated with the FAQ scores, also had significant associations with the Aβ_1–42_ concentrations.

To identify the associations between Aβ_1–42_ concentration level and the underlying gene patterns, we employed differential expression analysis and enrichment analysis by using the R script with the Limma package and DAVID v6.8. As shown in Fig. 4, 11 pathways were enriched based on the KEGG pathway map (see Supplementary Material 4 for the detailed characteristics). Among them, two pathways, including the calcium signaling pathway and the serotonergic synapse pathway, were found to be correlated with Aβ_1–42_.

## Discussion

Biomarkers have become increasingly important in predicting neurodegenerative processes in AD. As one of the newly discovered core neuropathological factors related to AD, Aβ peptides have attracted extensive attention. Aggregated Aβ peptides form plaques and fibrils and eventually lead to synapse loss and cell apoptosis [28]. In our study, only Aβ_1–42_ showed a significant correlation with patient status. Studies have proven that Aβ_1–42_ seems to be sensitive because it is more prone to accumulate than other isoforms [29]. Hence, the concentration of Aβ_1–42_ in CSF has been used to predict conversion to AD in many studies [30–32]. In our study, the predictive accuracy of Aβ_1–42_ was 0.769 in the training cohort, and 0.831 in the validation cohort, slightly lower than other predictors. The possible reason is that the progression of AD is unstable, and CSF biomarkers tend to gain more accuracy when assessed earlier in the process. A previous meta-analysis had shown that memory impairment and CSF abnormalities have approximately equal predictive power >4 years before the final diagnosis [33].

Neuroimaging is a critical technique in the clinical diagnosis of neurodegenerative conditions. However, it has always been used as a subjective or qualitative tool in traditional diagnostic situations. The development of computer science and image-processing technology has made it possible to quantify medical image information [34]. The use of newly developed radiomics approaches has pushed conventional research up to a translational level. This process includes converting images into a high-dimensional feature set and extracting significant biomarkers by selecting an algorithm to build a radiomics signature that provides reliable support for the identification and prediction of patient status. In this study, the Rad-sig based on LASSO-selected features had the C-indexes of 0.90 and 0.869 in the primary and validation cohorts, respectively. Surprisingly, we found that most selected features were located in the temporal cortex, such as the superior temporal sulcus and inferior temporal sulcus (see Supplementary Material 1). In fact, the temporal cortex has been referred to as one of most significant characteristics of the structural changes associated with AD progression [35, 36]. The above studies, as well as our findings, highlighted that structural changes in the temporal cortex may be more sensitive to AD progression, especially for MCI conversion. There were other features in the frontal and parietal cortices, which have also been identified in previous studies [37, 38].

The scores on the FAQ, as one of the widely used traditional neuropsychological scales, were the most accurate in predicting the MCI-to-AD progression (0.921 in primary cohort and 0.910 in validation cohort). Similarly, previous studies have found that neuropsychological scales have more predictive power than image features and CSF biomarkers [29, 39]. These results demonstrated that neuropsychological assessments are a very reliable and necessary predictor of AD progression.

However, individual predictors have significant challenges in predicting patient clinical outcomes. Abnormalities in Aβ peptides play a central role in AD neuropathology, but they might be less powerful as a single predictor. Neuroimaging has become an indispensable aid in the diagnosis of and research on neurodegenerative diseases, but cannot detect microscopic changes in AD progression. Although the neuropsychological scales had been proven to be a powerful predictive factor in MCI and AD, they had some shortcomings due to subjectivity. To address these issues, we constructed and validated a composite multipredictor nomogram for the estimation of the conversion risk of MCI patients from the ADNI. The proposed nomogram had better performance than the models based on the individual predictors alone. This result indicated that from a pragmatic perspective, using all the available data could make predictions that are more accurate and appear optimal in clinical practice.

In addition, we explored the associations between features from macro- to microperspective in the validation cohort. The FAQ scores, which represent the clinical manifestations and cognitive function of the MCI patients, were supposed to be correlated with the structural changes [40, 41]. As shown in Fig. 4, the FAQ scores indeed had strong correlations with five image features, including lh_S_intrapariet&P_trans_thickness, lh_S_temporal_sup_thickness, lh_G_temporal_inf_thickness, rh_G_temp_sup-Plan_polar_thickness and rh_S_temporal_inf_thickness. Interestingly, all of these features were also found to have strong associations with the concentrations of Aβ_1–42_ in the CSF. Several studies have demonstrated that the levels of Aβ in CSF are related to structural brain changes [42–45]. Combining our results with previous studies, we suspect that the concentrations of Aβ_1–42_ in the CSF might be correlated with cortex changes in the brain, especially in the temporal cortex.

Furthermore, we added a gene expression profile to further analyze the association between Aβ_1–42_ concentrations and the underlying gene pathways associated with AD. The differentially expressed genes were enriched in 11 gene pathways (see Supplementary Material 4 for detailed characteristics). We found only two pathways that were associated with Aβ_1–42_ concentrations by correlation analysis, including the calcium signaling pathway and serotonergic synapse pathway. A previous study found that Aβ influenced calcium homeostasis and impaired redox homeostasis in brain endothelial cells [46]. In particular, accumulated Aβ_1–42_ could disturb calcium homeostasis and lead to cell death [47, 48]. Convergent findings demonstrated that serotonin signaling could alter Aβ levels (i.e., regulation of amyloid precursor protein processing by serotonin signaling). Patients with AD who were treated with antidepressant drugs showed reduced Aβ_1–40_ and Aβ_1–42_ levels [49]. Consequently, treatment with selective serotonin reuptake inhibitors, as an anti-Aβ strategy, might be effective in preventing or halting Aβ plaque accumulation in the early stages of AD [50].

The limitations of this study included the lack of external validation and an in-depth exploration of pathological mechanisms. The patients in this study were all from the ADNI database, and an independent validation set is needed to acquire more evidence for the stability of the nomogram. Moreover, we lacked a pathological analysis of the associations between the predictive factors, and this work should be explored in the future.

In summary, our research constructed a powerful nomogram based on multiple factors to predict the conversion probability for MCI patients. Furthermore, we found significant associations between cognitive impairment, structural changes, Aβ level, and underlying gene patterns. With this study, we expect that the nomogram method will be fully developed to help clinical diagnosis and prediction. In addition, the associations from the macro- to microperspective would provide new information for the further exploration of neurodegeneration.

## Funding and disclosure

This work was supported by the National Natural Science Foundation of China [grant numbers 81671778, 81701787, and 81801789]. Data collection and sharing for this study were funded by the Alzheimer’s Disease Neuroimaging Initiative (ADNI) (National Institutes of Health Grant U01 AG024904) and DOD ADNI (Department of Defense award number W81XWH-12-2-0012). ADNI data are disseminated by the Laboratory for Neuro Imaging at the University of Southern California. All authors declare no potential conflicts of interest.

## Supplementary information

Supplementary Material 1

Supplementary Material 2

Supplementary Material 3

Supplementary Material 4
