# Supplementary material for: A multipredictor model to predict the conversion of mild cognitive impairment to Alzheimer’s disease by using a predictive nomogram
Source: Neuropsychopharmacology. 2019 Oct 21;45(2):358–66. doi: 10.1038/s41386-019-0551-0 (PMC6901533; doi:10.1038/s41386-019-0551-0)
Supplement: Supplementary file 1 — Supplementary Material 1 [file 41386_2019_551_MOESM1_ESM.docx]

1. **The list of the selected features:**

| **Symbol in Rad-sig formula** | **Feature name** | **Abbreviation** | **Coefficients** |
| --- | --- | --- | --- |
| X1 | Left hemisphere, superior part of the precentral sulcus, curvature index | lh_S_precentral-sup-part_curvind | -0.187119636 |
| X2 | Right hemisphere, intraparietal sulcus and transverse parietal sulci, folding index | rh_S_intrapariet&P_trans_foldind | -0.01013241 |
| X3 | Right hemisphere, superior temporal sulcus, folding index | rh_S_temporal_sup_foldind | -0.010835354 |
| X4 | Left hemisphere, superior frontal sulcus, mean curvature | lh_S_front_sup_meancurv | -4.241439695 |
| X5 | Left hemisphere, superior frontal sulcus, average thickness | lh_S_front_sup_thickness | -1.042841718 |
| X6 | Left hemisphere, intraparietal sulcus and transverse parietal sulci, average thickness | lh_S_intrapariet&P_trans_thickness | -0.84805223 |
| X7 | Left hemisphere, superior temporal sulcus, average thickness | lh_S_temporal_sup_thickness | -1.493027749 |
| X8 | Left hemisphere, inferior temporal sulcus, average thickness | lh_G_temporal_inf_thickness | -0.427696939 |
| X9 | Right hemisphere, planum polare of the superior temporal gyrus, average thickness | rh_G_temp_sup-Plan_polar_thickness | -0.613293755 |
| X10 | Right hemisphere, inferior temporal sulcus, average thickness | rh_S_temporal_inf_thickness | -0.906472439 |
| X11 | Left hemisphere, superior frontal sulcus, thickness standard deviation | lh_G_front_sup_thicknessstd | 2.904775915 |
| X12 | Left hemisphere, inferior part of the precentral sulcus, thickness standard deviation | lh_S_precentral-inf-part_thicknessstd | 0.226193734 |
| X13 | Left hemisphere, planum temporale or temporal plane of the superior temporal gyrus, thickness standard deviation | lh_G_temp_sup-Plan_tempo_thicknessstd | 1.902507618 |
| X14 | Right hemisphere, subcallosal area, subcallosal gyrus, thickness standard deviation | rh_G_subcallosal_thicknessstd | 0.095053004 |
| X15 | Left hemisphere, pericallosal sulcus, gray matter volume | lh_S_pericallosal_volume | -3.91E-05 |
| X16 | Left hemisphere, superior temporal sulcus, gray matter volume | lh_S_temporal_sup_volume | -4.50E-07 |
| X17 | Right hemisphere, inferior temporal sulcus, gray matter volume | rh_G_temporal_inf_volume | -2.41E-05 |

Reference:

Destrieux C, Fischl B, Dale A, et al. Automatic parcellation of human cortical gyri and sulci using standard anatomical nomenclature[J]. NeuroImage, 2010, 53(1):1-15.

1. **The Radiomics signature calculation formula:**

$Rad-sig= 10.57599202-0.187119636\times X1-0.01013241\times X2-0.010835354\times X3-4.241439695\times X4-1.042841718\times X5-0.84805223\times X6-1.493027749\times X7-0.427696939\times X8-0.613293755\times X9-0.906472439\times X10+2.904775915\times X11+0.226193734\times X12+1.902507618\times X13+0.095053004\times X14-3.91e^{-5}\times X15-4.5e^{-7}\times X16-2.41e^{-5}\times X17$
