# Supplementary material for: A multipredictor model to predict the conversion of mild cognitive impairment to Alzheimer’s disease by using a predictive nomogram
Source: Neuropsychopharmacology. 2019 Oct 21;45(2):358–66. doi: 10.1038/s41386-019-0551-0 (PMC6901533; doi:10.1038/s41386-019-0551-0)
Supplement: Supplementary file 2 — Supplementary Material 2 [file 41386_2019_551_MOESM2_ESM.docx]

**Spearman correlations of candidate predictors and the patients’ status in the training cohort (ranked by p-value from low to high):**

| Candidate predictors | Significance level (p) | Correlation coefficient (r) |
| --- | --- | --- |
| Demographic information |  |  |
| Age | 0.434 | 0.057 |
| Sex | 0.565 | -0.042 |
| Education level | 0.774 | 0.021 |
| Amyloid-Beta peptides in CSF aliquot samples |  |  |
| Aβ_1-42_ | $7.2072e^{-7}$ | -0.350 |
| Aβ_1-40_ | 0.145 | -0.106 |
| Aβ_1-38_ | 0.338 | -0.070 |
| Neuropsychological scales |  |  |
| FAQ | $6.1653e^{-28}$ | 0.686 |
| ADAS13 | $1.5028e^{-26}$ | 0.673 |
| ADAS11 | $2.4454e^{-24}$ | 0.650 |
| MMSE | $8.3779e^{-20}$ | -0.597 |
| NPI-Q | $6.6733e^{-7}$ | 0.361 |
| GDS | 0.400 | 0.061 |

Aβ_1-42_: Amyloid Beta 1-42; Aβ_1-40_: Amyloid Beta 1-40; Aβ_1-38_: Amyloid Beta 1-38; FAQ: Functional Activities Questionnaire; ADAS: Alzheimer's Disease Assessment Scale; MMSE: Mini-Mental State Examination; NPI-Q: Neuropsychiatric Inventory Questionnaire; GDS: Geriatric Depression Scale
