# Supplementary material for: A multipredictor model to predict the conversion of mild cognitive impairment to Alzheimer’s disease by using a predictive nomogram
Source: Neuropsychopharmacology. 2019 Oct 21;45(2):358–66. doi: 10.1038/s41386-019-0551-0 (PMC6901533; doi:10.1038/s41386-019-0551-0)
Supplement: Supplementary file 3 — Supplementary Material 3 [file 41386_2019_551_MOESM3_ESM.docx]

1. **The ROC curve in the primary and validation cohorts.**

**
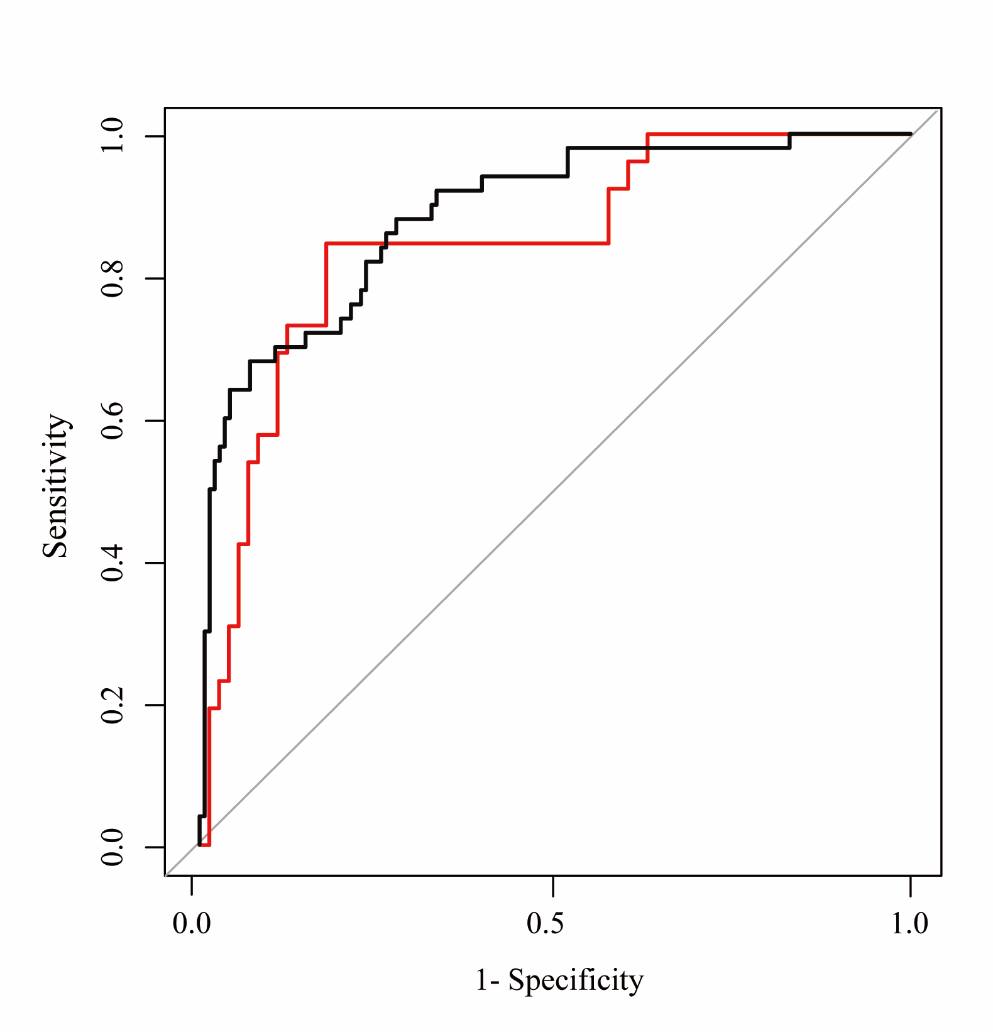
**

Sup-Figure 3.1 Assessment and validation of the image features by SVM classifier. The black ROC curve shows the assessment of selected image features in the primary cohort after 10-fold cross-validation. The red ROC curve shows the validation of elected image features in the validation cohort. The x-axis is the false positive rate (1-specificty) and the y-axis is the sensitivity.

**2. Performance of individual predictors and the multi-factors model in the primary cohort and validation cohort.**

| **Predictors** | **C-index in primary cohort [95% CI]** | **C-index in validation cohort [95% CI]** |
| --- | --- | --- |
| Neuropsychological scale (FAQ) | 0.921 [0.875, 0.967] | 0.910 [0.847, 0.973] |
| Radiomics signature (Rad-sig) | 0.90 [0.851, 0.949] | 0.869 [0.793, 0.945] |
| Amyloid-beta peptides in CSF (Aβ_1-42_) | 0.769 [0.695, 0.843] | 0.831 [0.749, 0.913] |
| Multi-predictors | 0.978 [0.960, 0.995] | 0.956 [0.919, 0.992] |
