# Supplementary material for: A multipredictor model to predict the conversion of mild cognitive impairment to Alzheimer’s disease by using a predictive nomogram
Source: Neuropsychopharmacology. 2019 Oct 21;45(2):358–66. doi: 10.1038/s41386-019-0551-0 (PMC6901533; doi:10.1038/s41386-019-0551-0)
Supplement: Supplementary file 4 — Supplementary Material 4 [file 41386_2019_551_MOESM4_ESM.docx]

**Enriched gene pathways based on differential expression analysis by using DAVID enrichment analysis.**

| Enriched pathways | No. of genes | Differential expression genes  between MCI_C and MCI_S | KEGG functional classification | P-value (corrected by Fisher exact) |
| --- | --- | --- | --- | --- |
| Salivary secretion | 87 | ATP1A2, ATP2B1, ADCY2, ADCY5, CALM1, CHRM3, PLCB4, PRH2, PRKG2, TRPV6 | Organismal systems and digestive system | 0.0018 |
| Calcium signaling pathway | 181 | ATP2B1, GNAL, ORAI3, ADCY2, CACNA1B, CALM1, CHRM3, NOS2, PLN, PLCB4, PTGER1, P2RX6, P2RX7, SPHK2, TRHR | Environmental information processing and signal transduction | 0.0041 |
| Pancreatic secretion | 93 | ATP1A2, ATP2B1, ADCY2, ADCY5, CHRM3, CELA3A, PNLIPRP2, PLA2G1B, PLCB4, SLC4A4 | Organismal systems and digestive system | 0.0032 |
| Ras signaling pathway | 226 | ELK1, FASLG, GNG4, HRAS, MET, RASSF5, SHC1, CALM1, EGF, FEGFR1, FLT4, IGF1R, MRAS, NFKB1, PAK6, PLA2G1B | Environmental information processing and signal transduction | 0.015 |
| Glucagon signaling pathway | 99 | ACACA, ADCY2, CALM1, CPT1C, G6PC, PCK1, PGAM1, PLCB4, PRKAG3 | Organismal systems and endocrine system | 0.015 |
| Insulin signaling pathway | 138 | ELK1, HRAS, SHC1, ACACA, CALM1, G6PC, HKDC1, PCK1, PRKAG3, PRKAR1B, TRIP10 | Organismal systems and endocrine system | 0.018 |
| Arginine and proline metabolism | 50 | AZIN2, CARNS1, CKMT1B, MAOB, NOS2, L3HYPDH | Amino acid metabolism | 0.012 |
| Gastric acid secretion | 73 | ATP1A2, ADCY2, ADCY5, CALM, CHRM3, PLCB4, SLC26A7 | Organismal systems and digestive system | 0.023 |
| Platelet activation | 130 | BTK, ADCY2, ADCY5, COL1A2, COL5A2, FGG, PLCB4, PTGIR, PTGS1, PRKG2, | Organismal systems and immune system | 0.03 |
| Serotonergic synapse | 111 | HTR1F, GNG4, HRAS, ADCY5, CACNA1B, CYP2C9, MAOB, PLCB4, PTGS1 | Organismal systems and nervous system | 0.029 |
| FoxO signaling pathway | 134 | FBXO32, FASLG, HRAS, SKP2, EGF, G6PC, IGF1R, PCK1, PRKAG3, RAG1 | Environmental information processing and signal transduction | 0.036 |
